# Clinical effects and safety of different transarterial chemoembolization methods for bridging and palliative treatments in hepatocellular carcinoma

**DOI:** 10.1007/s00432-021-03900-3

**Published:** 2022-01-25

**Authors:** Isabelle Mohr, Marie Vogeler, Jan Pfeiffenberger, Simon David Sprengel, Miriam Klauss, Boris Radeleff, Andreas Teufel, De-Hua Chang, Christoph Springfeld, Thomas Longerich, Uta Merle, Arianeb Mehrabi, Karl Heinz Weiss, Markus Mieth

**Affiliations:** 1 Internal Medicine IV, Department of Gastroenterology, Heidelberg University Hospital, Heidelberg, Germany; 2 Department of Radiology, Heidelberg University Hospital, Heidelberg, Germany; 3 Department of Diagnostic and Interventional Radiology, Sana Klinikum Hof, Hof, Germany; 4 Department of Gastroenterology and Hepatology, Mannheim University Hospital, Mannheim, Germany; 5 Department of Medical Oncology, Heidelberg University Hospital, National Center for Tumor Diseases (NCT), Heidelberg, Germany; 6 Department of Pathology, Heidelberg University Hospital, Heidelberg, Germany; 7 Department of General, Visceral and Transplantation Surgery, Heidelberg University Hospital, INF 110, 69120 Heidelberg, Germany; 8 Internal Medicine, Salem Hospital Heidelberg, Heidelberg, Germany; 9 Liver Cancer Center Heidelberg (LCCH), Heidelberg, Germany

**Keywords:** TACE, Chemoembolization, Liver cancer, HCC, Liver transplant

## Abstract

**Purpose:**

We assessed and compared clinical effects and safety endpoints of three methods of transarterial chemoembolization (TACE), conventional (cTACE), with drug-eluting beads (DEB-TACE), and with degradable starch microspheres (DSM-TACE), used in patients with hepatocellular carcinoma (HCC) in the bridging to liver transplant (LT) and the palliative setting.

**Methods:**

In our center, 148 patients with HCC underwent 492 completed TACE procedures between 2008 and 2017 (158 for bridging to LT; 334 for palliative treatment) which we analyzed retrospectively. Of these procedures, 348 were DEB-TACE, 60 cTACE, and 84 DSM-TACE.

**Results:**

The cTACE procedure revealed a significantly longer period of hospitalization (*p* = 0.02), increased occurrence of nausea (*p* = 0.025), and rise in alanine transaminase (ALT) levels (*p* = 0.001), especially in the palliative setting. In the bridging to LT cohort, these clinical endpoints did not reach statistical significance.

**Conclusions:**

The clinical safety of different TACE methods for HCC in both the palliative and the bridging to LT setting was equivalent. In the palliative setting, the cTACE procedure revealed an increased risk for adverse clinical effects such as nausea, elevation of ALT, and a prolonged period of hospitalization what might either be related to the systemic effects of the chemotherapeutic agent or to the differences in both collectives. Thus, further studies must be conducted on a larger number of TACE procedures to effectively explore the clinical side effects of the various TACE variants.

## Introduction

Management and prognosis of hepatocellular carcinoma (HCC) patients depends on tumor status, general health, and recent liver functional reserve (Okuda et al. 1985; Llovet et al. 1999; Marrero et al. 2005; Cabibbo et al. 2010). Curative treatments, such as resection, liver transplantation, or local ablation, are generally restricted to limited tumor mass (Bruix and Sherman 2005; Llovet et al. 2005, 2012). Transarterial chemoembolization (TACE) is currently considered the first line-therapy for intermediate-stage HCC patients (Llovet 2003; Llovet et al. 2008; Bruix and Sherman 2011), aiming for local tumor control (Arii et al. 2000; Ikai et al. 2004; Takayasu et al. 2006; Lee et al. 2012). Apart from its use in the palliative setting for intermediate and advanced tumor stages, TACE is applied as bridging treatment to liver transplantation (LT) to control local tumor growth and maintain the patient’s tumor load (Majno et al. 1997; Decaens et al. 2005; Porrett et al. 2006; Bruix 2011, Kollmann et al. 2017). The aim of TACE is to induce tumor necrosis of medium local application of high-dose chemotherapy and additional hypoxia (through vascular occlusion) (Zangos et al. 2007).

The efficacy of general TACE procedures has already been evaluated several times in patients with purely palliative therapy indications (Biselli et al. 2005; Llovet 2003; Takayasu et al. 2006). However, the benefits of TACE as bridging therapy for liver transplantation have been studied to a much lesser extent, with unambiguous results (Decaens et al. 2005; Fujiki et al. 2011; Holowko et al. 2015; Majno et al. 1997; Porrett et al. 2006). Currently, different variants of TACE are in use. These include conventional TACE (cTACE), drug-eluting bead TACE (DEB-TACE), and TACE with degradable starch microspheres (DSM-TACE). In cTACE, a mixture of the chemotherapeutic agent and an embolizing material is usually administered at the beginning of TACE procedure, after which a single dose of the embolisate is administered until the blood flow in the artery supplying the tumor ceases (Gruber-Rouh et al. 2018). The cTACE procedure is carried out with different chemotherapy and embolising materials depending on the clinic and the scheme used (Lencioni and Llovet 2010). The most commonly used embolizing material is Lipiodol (Vogl and Gruber-Rouh 2019). It plays a central role in cTACE as it is simultaneously used as a carrier substance for the chemotherapy drug, as an X-ray contrast agent for marking the tumor and as an embolizing material (Liapi and Geschwind 2011). The most widely used chemotherapeutic drug for cTACE worldwide is doxorubicin (Lencioni et al. 2013)—although agents such as epirubicin, mitomycin, cisplatin, and miriplanin are also used (Vogl and Gruber-Rouh 2019). The different application of cTACE, with regard to the technique and the therapy plan, limits the comparability of cTACE (Lencioni et al. 2013). The DEB-TACE procedure describes the intraarterial application of beads loaded with chemotherapeutically active substances to achieve a continuous release of these substances in Vogl and Gruber-Rouh (2019). These beads are available in different sizes and, in contrast to cTACE, lead to a longer dwell of the chemotherapeutic agent in the tumor, with at the same time less systemic effects (Poon et al. 2007; Varela et al. 2007; Vogl and Gruber-Rouh 2019). This is due to the lack of a time gap between the application of the chemotherapeutic agent and the embolisate as the beads act simultaneously as both thereby making an additional application of embolizing substances unnecessary. DC Beads® (non-biodegradable, polyvinyl alcohol-laden microspheres loaded with doxorubicin) are used most commonly for performing DEB-TACE procedures (Song and Kim 2017). Due to the lower plasma concentration of the chemotherapeutic agent in a DEB-TACE, significantly fewer drug-related adverse reactions could already be observed compared to cTACE (Varela et al. 2007; Lencioni et al. 2012). Nevertheless, the superiority of DEB-TACE over cTACE continues to be questioned due to insufficient randomized controlled studies (Facciorusso 2018). The most modern variant of TACE uses more biodegradable particles (degradable starch microspheres = DSM), such as EmboCept® S particles (Caine et al. 2017). As DSM-TACE is a recent development, it has been the topic of a few studies, some of which found both a favourable secondary level profile and a sufficient effectiveness of biodegradable particles in TACE (Kirchhoff et al. 2007; Orlacchio et al. 2015; Schicho et al. 2017). However, data comparing the efficacy of the different TACE methods used in the bridging to transplant with those used in the palliative collective are rare. Here, we retrospectively assessed and compared the clinical safety and efficiency of the TACE variants used for bridging to transplant and in palliative procedures in HCC patients.

Although the results of sorafenib therapy in combination with other therapies, such as TACE, were positive in various meta-analyses (time-to-progress (TTP)), they were less so for overall survival (Zeng et al. 2016; Li et al. 2018; Wang et al. 2018). As a multikinase inhibitor, sorafenib interferes with the proliferation mechanism of tumor cells and their angiogenesis while increasing apoptosis (Chang et al. 2007). However, due to its systemic application, as opposed to the local application within the framework of a TACE, side effects, such as diarrhea, nausea, vomiting, and the hand–foot–skin syndrome ensue (Li et al. 2015).

## Materials and methods

### Study design

**Fig. 1:**
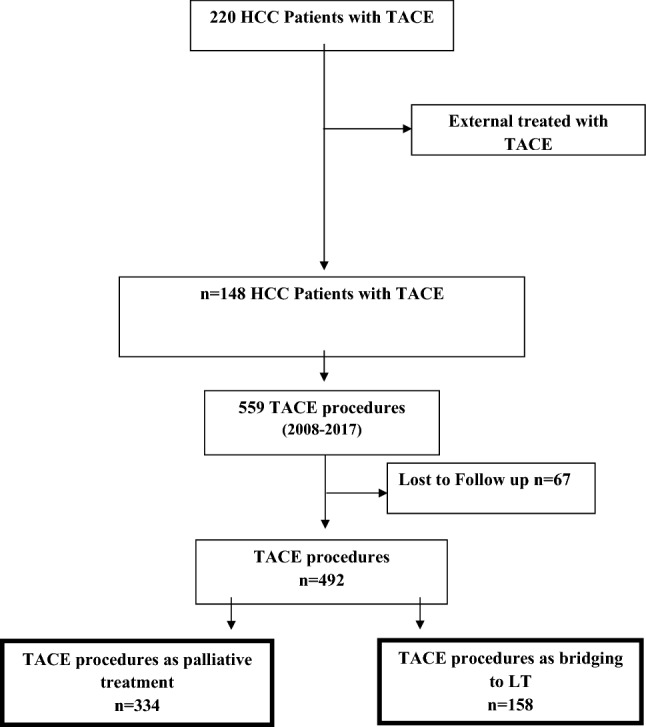
Flow chart showing the selection of transarterial chemoembolization (TACE) procedures as well as the final number of included TACE procedures of different subgroups

The retrospective cohort study was conducted in a tertiary care center (Heidelberg University Hospital) and was approved by the institutional review board (IRB). Data collection was based on chart review of patients with established diagnosis of HCC, based on the EASL (European Association for the Study of the Liver) criteria, who had undergone at least one TACE procedure as a therapy for HCC between 2008 and 2017 in our center. In this period, 148 patients with HCC underwent 492 completed TACE procedures (158 for bridging to transplant; 334 for palliative treatment; Fig. 1). Indication for TACE procedure was definded by multidisciplinary tumor board. The board’s treatment approach followed the current EASL–EORTC (European Organization for Research and Treatment of Cancer) Clinical Practice Guidelines (2012) in patients who had unresectable lesions and for whom other ablative therapies were not suitable. Patients who had been diagnosed with BCLC (Barcelona Clinic Liver Cancer) score (stage A, C, or D, but were unable or unwilling to receive the proposed therapy (e.g., LT, radiofrequency ablation (RFA), sorafenib) were also eligible for TACE therapy. For patients on the LT list, TACE was considered a standard bridging treatment. All decisions on the type of TACE treatment and modality of beads (cTACE vs. DSM-TACE or DEB-TACE vs. DSM-TACE) to be used in the patients were at the interventionalist's discretion.

### Subgroup definition

Each TACE procedure of the included patients was categorized into two different subgroups, depending on their treatment plan at the time of TACE therapy: bridging to transplant or palliative therapy. Both collectives were split up into the different TACE methods: cTACE, DEB-TACE, and DSM-TACE. Within the palliative collective 244 DEB-TACE, 28 cTACE, and 62 DSM-TACE procedures were performed. In the bridging cohort 104 DEB-TACE, 32 cTACE, and 22 DSM-TACE procedures were conducted. The bridging to LT data set included all interventions in which patients were enrolled on the transplant waiting list at the time of TACE, regardless of whether the LT was performed afterwards. The palliative data set consisted of interventions performed in patients who did not meet the criteria for a LT at the time of TACE.

### Statistics

Statistical analysis was performed using the SPSS-25 software (IBM, Germany). The two-tailed chi-squared test was employed to compare the categorical data from different TACE variants used in the bridging and palliative data sets. Statistical comparative calculation between the subgroups for continuous endpoints was performed using the Kruskal–Wallis test. Statistical significance was set at *p* value < 0.05 in two-tailed tests. Categorial endpoints in the study were clinical symptoms, such as nausea, vomiting, abdominal pain, fever, antibiotic treatment, rise of transaminases, and period of hospitalization.

## Results

### Patient characteristics at TACE procedures

A total of 492 TACE sessions were included in this study (158 bridging/334 palliative TACE sessions). As expected, due to listing criteria, patients in the bridging cohort were younger, had limited tumor spread, and different tumor properties, such as less frequent portal or hepatic vein infiltration and no extrahepatic tumor manifestation. The median age at the time of the various TACE procedures in the curative collective was about 58 years, whereas the median age in patients treated with palliative care was about 68 years at the time of the TACE session.

Similarly, the TACE collectives differed in terms of the number of tumorous lesions. Most patients showed multilocular HCC findings in both the curative and palliative collectives at the time of a TACE procedure. The maximum diameter of the HCC lesions at the time of a TACE session also differed significantly within both collectives. Patients undergoing TACE in palliative intent had a median lesion size that was twice as large in diameter as that in the bridging cohort.

Conventional TACE was performed more than twice as often in the curative cohort as it was in the palliative intent. However, the DEB-TACE and DSM-TACE procedures were carried out more frequently in the palliative intent cohort than in the curative one. In addition, noticeable was a significantly more frequent discontinuation of therapy due to adverse drug reactions/adverse events (AE) or death. There was no discontinuation of therapy due to AE or death in the bridging cohort. In the palliative collective, adverse or general reactions, which included both laboratory chemical and image morphological abnormalities after TACE as well as subjective and indirect parameters, occurred in all TACE variants with a relative frequency of over 90%. The therapy was discontinued due to AE or death in 21.9% of all cases (Table 1).

### Results in the palliative data set

A total of 244 procedures were carried out using the eluting beads as the active ingredient. We found that 28 sessions were conducted using cTACE with Carboplatin or Doxorubicin as the chemotherapeutic agent in combination with Lipiodol, which is only half as common as that in the bridging data set and 62 TACE sessions were conducted using DSM-TACE. In the palliative group, 73 (21.9%) TACE sessions were performed in patients who finally discontinued TACE therapy (and received no further local therapy) because of AE or death, whereas in the bridging data set none of the patients discontinued the TACE therapy (Table 2).

**Table 1:** Baseline characteristics of the bridging and palliative data sets

Baseline characteristics	Bridging data set		Palliative data set		
	N (%)	Total (N)	N (%)	Total (N)	p
**Number of patients**	**61 (100)**		**87 (100)**		
**Age (at first TACE). Years (median. range)**	58 (28–69)	61	68 (49–88)	87	** < 0.001**
**Gender**					
Male	47 (77..0)	61	71 (81.6)	87	0.497
Female	14 (23.0)		16 (18.4)		
A	40 (65.6)	61	17 (19.5)	87	** < 0.001**
B	9 (14.8)		34 (39.1)		
C D	4 (6.6) 8 (13.1)		29 (33.3) 7 (8.0)		
Child–Pugh class					
A	32 (52.5)	61	55 (63.2)	87	0.370
B	21 (34.4)		25 (28.7)		
C	8 (13.1)		7 (8.0)		
Cirrhosis	58 (95.1)	61	68 (78.2)	87	**0.004**
Number of tumor nodules					
< 1 > 1	28 (45.9) 33 (54.1)	61	25 (28.7) 62 (71.3)	87	**0.032**
Maximal diameter, cm (median, range)	2.5 (1.2–4.9)	61	4.7 (1.0–16.5)	87	** < 0.001**
AFP, ng/ml					
< 400 > 400	55 (93.2) 4 (6.8)	59	70 (82.4) 15 (17.6)	85	0.058
Portal vein thrombosis	0 (0.0)	61	7 (8.0)*	87	**0.023**
Vena cava thrombosis	0 (0.0)	61	1 (1.1)	87	0.401
Liver vein thrombosis	0 (0.0)	61	2 (2.3)	87	0.233
Extrahepatic spread	0 (0.0)	61	8 (9.2)	87	**0.015**
Esophageal varices	40 (65.6)	61	36 (41.1)	87	**0.004**
Etiology					
Viral	23 (37.7)	61	33 (37.9)	87	0.270
Alcohol abuse	19 (31.1)		26 (29.9)		
Viral + alcohol	7 (11.5)		6 (6.9)		
NASH	0 (0)		2 (2.3)		
Cryptogenic	4 (6.6)		13 (14.9)		
Autoimmune	4 (6.6)		1 (1.1)		
Others	4 (6.6)		6 (6.9)		

Statistically significant p values are in bold (*p* < 0.05)

*TACE*  transarterial chemoembolization, *BCLC* barcelona clinic liver cancer, *AFP* alpha fetoprotein

*Subtotal thrombosis; patients were switched to systemic therapy after confirmation of diagnosis

**Table 2:** Comparison between transarterial chemoembolization (TACE) therapy characteristics in the bridging vs. palliative data sets

Characteristics of TACE	Bridging data set		Palliative data set		
Therapy	*N* (%)	(*N*)	*N* (%)	(*N*)	*p*
Number of patients Number of TACE procedures in general	**61 (100)** 158 (100)		**87 (100)** 334 (100)	561	
Completed TACE-procedures per patient (median, range)	3 (1–10)	61	3 (1–10)	87	0.072
Time from first completed to last completed TACE (except patients with only one TACE-procedure), months (median, range)	3.5 (0.9–35.0)	48	8.0 (0.9–58.6)	71	**0.009**
Change to systemic therapy	7 (11.5)	61	10 (11.5)	87	0.997
Progress	22 (36.1)	61	59 (67.8)	87	** < 0.001**
Time to progress, months (median, 95% CI)	13.8 (11.5–16.2)	61	9.7 (6.0–13.4)	87	0.054
Category of TACE					
DEB Conventional Biodegrad Others	39 (63.9) 9 (14.8) 5 (8.2) 8 (13.1)	61	55 (63.2) 3 (3.4) 11 (12.6) 18 (20.7)	87	0.058
TACE therapy discontinuation	21 (34.4)	61	79 (90.8)	87	** < 0.001**
Reason of therapy discontinuation					
Death Progress Technical AE Others	0 (0) 6 (28.6) 9 (42.9) 0 (0) 6 (28.6)	21	12 (15.2) 24 (30.4) 14 (17.7) 7 (8.9) 22 (27.8)	79	**0.047**
Other therapy received after therapy discontinuation	16 (76.2)	21	45 (57.0)	79	0.108
Additional TACE received despite progress	18 (81.1)	22	46 (78.0)	59	0.705
Therapy type after TACE					
Sorafenib SIRT RFA IRE Others/var	6 (37.5) 1 (6.3) 3 (18.8) 2 (12.5) 4 (25.0)	16	26 (57.8) 3 (6.7) 3 (6.7) 3 (6.7) 10 (22.2)	45	0.523
Deceased	19 (31.1)	61	55 (63.2)	87	** < 0.001**
Median survival time after first completed TACE-procedure (median, 95% CI)	Not reached (*)	61	22.5 (15.7–29.4)	87	** < 0.001**

Statistically significant p values are in bold (*p* < 0.05)

*SIRT* selective internal radiotherapy, *RFA* radio frequency ablation, *IRE* irreversible electroporation

#### Tumor response

The radiological response of the tumor to TACE therapy was calculated by a subdivision of the mRECIST Score (modified Response Evaluation Criteria in Solid Tumors). Highest partial remission results in staging after 4–6 weeks of TACE therapy were achieved with 21% of the cases in the cTACE group, whereas stable disease was percentual nearly similar in DEB-TACE and in DSM-TACE (64% vs. 70%). Progress up to 6 weeks after a TACE meeting was most common after DEB-TACE (22.5%) and more rare after DSM-TACE (12.9%) even if this did not involve any statistical significance.

#### Clinical side effects

Patients receiving cTACE were significantly more likely (22%) to report post-interventional nausea than patients treated with DEB-TACE (8.1%) or DSM-TACE (4.9%; Kruskal Wallis Test: *p* = 0.025; see Table 3). Although the occurrence of post-interventional abdominal pain did not reveal a significant difference in the various TACE variants, it is nevertheless noticeable that this undesirable drug effect also occurred most frequently after cTACE treatments in about 41% of the cases (Table 3). The same applies to the occurrence of post-interventional fever, as well as vomiting, and increase in transaminases after the cTACE session. Nausea and vomiting still were more often detected under systemic therapy (47.1%). Vascular complications, such as thrombosis or fistulas, did not occur after cTACE, whereas these could be observed sporadically after both DSM-TACE and DEB-TACE procedures Portal vein thrombosis is generally considered to be a contra-indications against TACE. In the seven patients of the palliative cohort, portal vein thrombosis was a subtotal thrombosis and occurred under TACE therapy, possibly as a progress of the tumor under interventional therapy. After diagnosis was confirmed via CT scan, no further TACE was performed. Thus, the patients were switched to a systemic chemotherapy. Antibiotic treatment due to an increased post-interventional CRP (c-reactive protein) was observed in about 18–19% of all TACE procedures, regardless of which TACE variant was used.

**Table 3:** Outcome parameters in the palliative data set

Outcome parameters	DEB-TACE	cTACE	DSM-TACE	
Palliative data set	*N* (%)	*N* (%)	*N* (%)	*P*
Number of TACE sessions	**244 (100)**	**28 (100)**	**62 (100)**	
Adverse events in general	211 (89.8)	28 (100)	60 (96.8)	0.052
Abdominal pain	65 (27.8)	11 (40.7)	11 (18.0)	0.076
Nausea	19 (8.1)	6 (22.2)	3 (4.9)	**0.025**
Fever	43 (18.4)	6 (22.2)	10 (16.4)	0.808
Vomiting	7 (3.0)	2 (7.4)	0 (0)	0.142
Increase of AST	181 (83.0)	19 (95.0)	49 (80.3)	0.307
Increase in UI/l (Median, SD)	77.0 (308.3)	232.0 (248.1)	130.0 (318.9)	0.053
Increase of AST > 5 × ULN	66 (28.0)	12 (50.0)	20 (32.8)	0.075
Increase of ALT	171 (78.8)	19 (95.0)	46 (75.4)	0.167
Increase in UI/l (Median, SD)	39.0 (217.0)	92.0 (319.3)	52.5 (114.5)	0.057
Increase of ALT > 5 × ULN	24 (9.9)	9 (36.0)	7 (11.3)	**0.001**
Portal vein fistula	9 (3.8)	0 (0)	1 (1.7)	0.441
GI-Ulcer	4 (1.7)	0 (0)	0 (0)	0.482
Cholecystitis	2 (0.8)	0 (0)	2 (3.4)	0.238
Abscess	7 (3.0)	1 (3.7)	0 (0)	0.389
Increase of creatinine	124 (56.9)	12 (63.2)	41 (68.3)	0.263
Increase in mg/dl (Median. SD)	0.08 (0.22)	0.17 (1.54)	0.07 (0.09)	**0.022**
Antibiotic treatment (AB)				
Periinterv	23 (9.8)	4 (14.8)	5 (8.2)	0.158
Postinterv	10 (4.3)	4 (14.8)	1 (1.6)	
After CRP ↑	81 (34.6)	9 (33.3)	23 (37.7)	
Without AB	120 (51.3)	10 (37.0)	32 (52.5)	
Analgesic therapy post TACE	201 (82.7)	26 (92.9)	47 (78.3)	0.243
Hospital stay, days (median, range)	4 (2–25)	5 (3–15)	4 (2–28)	**0.020**
Staging 4–6 weeks after TACE				
PR	25 (10.2)	6 (21.4)	7 (11.3)	0.242
SD	156 (63.9)	14 (50.0)	43 (69.4)	
PD	55 (22.5)	6 (21.4)	8 (12.9)	
No staging	8 (3.3)	2 (7.1)	4 (6.5)	

Statistically significant p values are in bold (*p* < 0.05)

*TACE* transarterial chemoembolization, *cTACE* conventional transarterial chemoembolization, *DEM-TACE* drug-eluting bead transarterial chemoembolization, *DSM-TACE*  transarterial chemoembolization with degradable starch microspheres, *AST* aspartate transaminase, *ALT*  alanine transaminase, *SD* stable disease, *CR*  complete remission, *PR*  partial remission, *SD*  stable disease, *PD* progressive disease

#### Laboratory effects

Similarly, some post-interventional laboratory values differed to a significant extent depending on which TACE variant was used. This includes, for example, the post-interventional increase in creatinine, which was observed to be highest after cTACE (Δ 0.17; Table 3). Nevertheless, this is conditional to the amount of injected contrast agent, what was not analyzed. Definitive acute or chronic renal failure was not observed yet.

It was significantly more likely for a five-times increase above the upper normal limit (> 5 × ULN) in the alanine transaminase (ALT) levels of patients following a cTACE procedure (36%; *p* = 0.001). This was neither the case for aspartate transaminase (AST) increase nor for AST > 5 × ULN.

#### Period of hospitalization

Furthermore, the median hospital stay after a DEB-TACE and a DSM TACE procedure was about 4 d, whereas patients who underwent cTACE spent 5 days in hospital. Nevertheless, these differences reached statistical significance in the Kruskal–Wallis test (*p* = 0.02; Table 3).

### Results in the bridging data set

Table 4 lists the clinical outcome parameters depending on the TACE variant that was performed in the bridging data set. In the curative collective, 104 procedures were carried out using active ingredient-eluting beads (DEB-TACE). A total of 32 sessions were conducted using cTACE and 22 using biodegradable particles (DSM-TACE).

**Table 4:** Outcome parameters for the bridging data set

Outcome parameters	DEB-TACE	cTACE	DSM-TACE	
bridging data set	*N* (%)	*N* (%)	*N* (%)	*p*
Number of TACE sessions	**104 (100)**	**32 (100)**	**22 (100)**	
Adverse events in general	90 (90.9)	29 (93.5)	21 (95.5)	0.732
Abdominal pain	33 (32.7)	11 (34.4)	7 (31.8)	0.977
Nausea	11 (10.9)	6 (18.8)	3 (13.6)	0.510
Fever	8 (7.9)	2 (6.3)	2 (9.1)	0.923
Vomiting	3 (3.0)	2 (6.3)	0 (0)	0.429
Increase of AST	72 (75.0)	22 (78.6)	17 (77.3)	0.917
Increase in UI/l (Median, SD)	48.0 (742.8)	39.0 (122.3)	66.0 (198.9)	0.824
Increase of AST > 5 × ULN	22 (21.6)	4 (13.3)	4 (18.2)	0.598
Increase of ALT	65 (67.7)	21 (75.0)	18 (81.8)	0.372
Increase in UI/l (Median, SD)	26.0 (526.8)	22.0 (41.4)	21.0 (87.8)	0.945
Increase of ALT > 5 × ULN	9 (8.8)	1 (3.4)	3 (13.6)	0.425
Portal vein fistula	1 (1.0)	3 (9.4)	0 (0)	**0.027**
GI-Ulcer	3 (3.0)	0 (0)	0 (0)	0.451
Cholecystitis	4 (4.0)	0 (0)	0 (0)	0.340
Increase of creatinine	61 (62.9)	15 (53.6)	17 (77.3)	0.224
Increase in mg/dl (Median, SD)	0.07 (0.25)	0.06 (0.07)	0.13 (0.26)	**0.005**
Antibiotic treatment (AB)				
Periinterv	6 (5.9)	3 (9.4)	2 (9.1)	0.861
Postinterv	3 (3.0)	1 (3.1)	2 (9.1)	
After CRP ↑	19 (18.8)	6 (18.8)	4(18.2)	
Without AB	73 (72.3)	22 (68.8)	14 (63.6)	
Analgesic therapy post TACE	75 (73.5)	26 (81.3)	19 (86.4)	0.349
Hospital stay, days (median, range)	4 (2–22)	4 (2–8)	4 (2–16)	0.688

Statistically significant p values are in bold (*p* < 0.05)

*ULN* upper limit of normal, *UI* international unit, *SD* standard deviation

#### Tumor response

The radiological response of the tumor to TACE showed highest partial remission results in staging 4–6 weeks after TACE therapy, with 25% occurring in the DEB-TACE group, whereas stable disease was percentual nearly similar in DEB- and DSM-TACE (60% vs. 59%, respectively) and even more frequent in cTACE (66%). Progress up to 6 weeks after a TACE procedure was most common in the DSM-TACE group (23%) and most rare in DEB-TACE (8.7%). These results did not reach any statistical significance. 42 of the 61 patients underwent transplantation. The pathological response of the explanted liver revealed in 17 cases no vital tumor cells, while in 25 cases, vital cells were still detectable. Compared to the radiological response, a progress under therapy was detected in 22 patients, which nearly fits in line with the pathologists examinations.

#### Clinical side effects

Patients receiving cTACE treatments were more likely to report post-interventional nausea/vomiting and abdominal pain than those treated with DEB-TACE or DSM-TACE. In contrast, post-interventional fever was reported more frequently in patients receiving DSM-TACE (9.1%). Antibiotic treatment due to an increased post-interventional CRP was observed in about 33–38% of all TACE procedures, regardless of which TACE variant was used.

#### Laboratory effects

There were no significant differences in the laboratory-determined increase of transaminases or the increase in creatinine levels between the different TACE procedures.

#### Period of hospitalization

Median hospital stay in all TACE groups was about 4 d. Therefore, the period of hospitalization revealed no significance in the Kruskal–Wallis test (*p* = 0.688).

## Discussion

The determination of the most acceptable and prognostically most favorable TACE procedure depending on the patient’s condition and the treatment indication is a central point in the therapy of patients with HCC. In this study, significant differences were observed for the bridging TACE collective in terms of post-interventional AE (clinical or laboratory AE), tumor response, and period of hospitalization. Nevertheless, this study has some limitations due to patient cohorts (bridging vs. palliative collective) and due to variant TACE procedures (different particle size, different area of embolization, amount of embolisate, selective vs. unselective TACE).

As already expected, patient cohorts are heterogeneous which is consistent with intend of curative, respectively, palliative therapy concept. Comparing the different TACE procedures, number of cTACE procedures is less in comparison to the amount of DEB- and DSM-TACE. There might be a selection bias using c-TACE/DEB-TACE in fitter patients and DSM-TACE in cases of advanced tumor size.

### Bridging TACE cohort

The safety of the various TACE variants for the curative TACE collective or for patients receiving TACE as bridging therapy has so far been investigated to an insufficient extent. Although various studies have shown the evidence of a superior side effect profile with regard to various parameters in a DEB-TACE procedure compared to that with cTACE, these studies were mostly related to patients with an advanced HCC or to a palliative therapy indication (Lammer et al. 2010; Puchol 2011; van Malenstein et al. 2011; Recchia et al. 2012; Golfieri et al. 2014; Kloeckner et al. 2015; Zou et al. 2016; Lee et al. 2017; Song and Kim 2017; Melchiorre et al. 2018). After cTACE, an increase in abdominal pain, nausea, and vomiting was observed in our study, which, however, this did not reach significance. The post-interventional increase of liver enzymes such as AST and ALT did not offer a significant difference in the analysis of the curative collective. Nevertheless, the average ALT and AST levels after a DEB-TACE increased to a higher degree than those after cTACE. This is in contrast to previous studies which observed an increase in liver parameters mainly or to a higher extent after cTACE procedures (Lammer et al. 2010; Recchia et al. 2012; Golfieri et al. 2014). However, these studies do not separate TACE intervention into curative and palliative patient collectives. Since DSM-TACE is generally used in patients with a palliative purpose (Kirchhoff et al. 2007; Iezzi et al. 2016, 2019), its adverse reactions in patients with curative intention has not been sufficiently investigated. Although Schicho et al. (2017), Gruber-Rouh et al. (2018), and Orlacchio et al. (2018) included patients who underwent DSM-TACE with curative intent in their studies, they did not analyze them separately for the occurance of AE (Schicho et al. 2017; Gruber-Rouh et al. 2018; Orlacchio et al. 2018). Orlacchio et al. (2015) investigated DSM-TACE procedures with a view to assessing their safety and potential side effects and compared these with other TACE procedures by undertaking a literature research and found that DSM-TACE provides a similar level of security compared to cTACE and DEB-TACE for a curative collective; however, this requires further investigation due to the small number of cases examined in the study by Orlacchio et. al. (2015). In contrast to previous studies, in our analysis of the curative collective, the DSM-TACE showed a significantly higher increase in creatinine levels after the procedure compared to that after treatment with other TACE variants (Table 4). The DSM-TACE procedure has been described as a tolerable and low-impact TACE variant, and is particularly suitable for patients with impaired hepatic function due to its low systemic mode of action (Niessen et al. 2014; Schicho et al. 2017; Orlacchio et al. 2018; Iezzi et al. 2019). As with the assessment of the various side effects, there are particularly no studies that take both factors into account: the curative indication along with the kind of TACE variant used. Although there are several studies that compare the effects of a cTACE procedure to those of a DEB-TACE one, they do not explicitly refer to TACE as a curative intention and, therefore, do not compare palliative and curative TACE procedures separately.

The effectiveness of a DSM-TACE in comparison with the other TACE variants has been studied only in a few studies, primarily without differentiation between the respective therapy indications (Kirchhoff, et al. 2007; Niessen et al. 2014; Gruber-Rouh et al. 2018). Similarly, Orlacchio et al. (2015) offered not only a comparable safety, but also a comparable effectiveness of a DSM-TACE compared to the other TACE variants in relation to the curative objective of therapy (Orlacchio et al. 2015). Both studies seem to be correlated to our findings, but a definitive interpretation should be avoided due to the limited number of cases.

### Palliative TACE cohort

In contrast to those in the curative TACE collective, several studies with comparative analyses between the different TACE variants have already been published in the palliative setting. Our data also compared the treatment intention between the different TACE variants. In our analysis, only cTACE showed a significantly more frequent occurrence of various post-interventional side effects. After cTACE, nausea was the most frequently reported side effect. In the palliative cohort, nausea was significantly more often reported in patients receiving cTACE (*p* = 0.025). This might be associated with the systemic effect of the chemotherapeutic substances used for cTACE, e.g., doxorubicin. Other parameters such as abdominal pain, vomiting, and fever were also most common after cTACE, but did not reach significance in the statistical analysis. Similarly, creatinine was significantly increased in cTACE in comparison to other TACE variants. A significant post-interventional ALT increase (> 5 × ALT) could also be observed more often after a cTACE procedure.

In addition, patients with a cTACE procedure spent on average a day longer in the hospital than the patients receiving a different TACE variant. A certain advantage of a DSM-TACE or a DEB-TACE over cTACE in terms of safety has already been identified in several studies (Lammer et al. 2010; Puchol et al. 2011; van Malenstein et al. 2011; Recchia et al. 2012; Jang et al. 2015; Lee et al. 2017; Song and Kim 2017; Melchiorre et al. 2018). In most of these studies, the general comparison of cTACE and DEB-TACE was made, whereas the comparative assessment of all three TACE variants was hardly included in any study. Although our results showed no significance in terms of post-interventional AST or ALT levels, our higher AST and ALT values for cTACE on average compared to DEB-TACE are consistent with several studies that also observed a more pronounced increase in these parameters after cTACE (Lammer et al. 2010; Sacco et al. 2011, 2017; Golfieri et al. 2014).

However, a direct comparison of DSM-TACE with the other TACE variants has not been made yet. Thus, an advantage of the DSM-TACE over other TACE variants in terms of the spectrum of side effects remains without clear evidence. The results in the palliative collective offer a certain advantage of the DSM-TACE and DEB-TACE procedures over a cTACE. Although the case numbers in the palliative collective are greater than those in the bridging collective, further large-scale studies are needed to detect a clear advantage of a particular TACE variant and to analyze the effectiveness and safety of different TACE variants depending on the respective therapy indications.

## Conclusions

The treatment of patients suffering from HCC is also difficult to follow, even with currently more or less clear recommendations. The use of TACE therapy has already become a clear priority in the treatment of these patients, although the question of the therapy intention and the correct TACE variant for specific patient or tumor properties still has not been clarified clearly. Our aim was to identify significant differences in the different collectives (palliative vs. curative), to assess the clinical outcome depending on the TACE variant used, and to assess the applicability of different classification systems in the respective collectives. Based on our descriptive analysis and the significant differences between the curative and palliative collectives, both in terms of patient characteristics and course parameters, further clinical decisions should be prognostic analyses and the applications of classification systems should always be carried out depending on the respective therapy indication. In the curative collective, all TACE variants proved to have a similarly safety with regard to AE studied, so that the preference of a particular TACE procedure cannot be specified. In the palliative collective, there was a tendency to perform cTACE in relation to the adverse reaction profile. However, with relatively small numbers of cases in the analysis of the various TACE variants, further studies are needed to make a definitive statement in this regard.

## Data Availability

All data for the study are provided along with the article.

## Code availability

NA.
